# Micelle Enhanced Fluorimetric and Thin Layer Chromatography Densitometric Methods for the Determination of (±) Citalopram and its *S* – Enantiomer Escitalopram

**DOI:** 10.4137/aci.s2274

**Published:** 2009-04-07

**Authors:** Elham A. Taha, Nahla N. Salama, Shudong Wang

**Affiliations:** 1 National Organization for Drug Control and Research (NODCAR), 6-Abu Hazem Street, Pyramids Ave. P.O. Box 29, 35521 Giza, Egypt; 2 School of Pharmacy and Centre for Biomolecular Sciences, University of Nottingham, University Park, NG7 2RD, U.K

**Keywords:** citalopram, escitalopram, micelle fluorimetry, spiked plasma, thin layer chromatography densitometry, chiral selectors

## Abstract

Two sensitive and validated methods were developed for determination of a racemic mixture citalopram and its enantiomer *S*-(+) escitalopram. The first method was based on direct measurement of the intrinsic fluorescence of escitalopram using sodium dodecyl sulfate as micelle enhancer. This was further applied to determine escitalopram in spiked human plasma, as well as in the presence of common and co-administerated drugs. The second method was TLC densitometric based on various chiral selectors was investigated. The optimum TLC conditions were found to be sensitive and selective for identification and quantitative determination of enantiomeric purity of escitalopram in drug substance and drug products. The method can be useful to investigate adulteration of pure isomer with the cheap racemic form.

## Introduction

**Figure 1.**
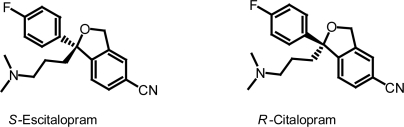
Chemical structure of citalopram enantiomers.

Citalopram (Fig. 1) a selective serotonine re-uptake inhibitor (SSRI), has been used for the treatment of depression, social anxiety disorder, panic disorder and obsessive-compulsive disorder.^1^–^3^ Citalopram is sold as a racemic mixture, consisting of 50% *R*-(−)-citalopram and 50% *S*-(+)-citalopram. As the *S*-(+) enantiomer has the desired antidepressant effect^4^ it is now marketed under the generic name of escitalopram. It has been shown that the *R*-enantiomer present in citalopram counteracts the activity of escitalopram. Citalopram and escitalopram have demonstrated different pharmacological and clinical effects.^5^

A number of techniques including spectrophotometric,^6^,^7^ fluorimetric,^8^,^9^ electrochemical,^10^ chromatography^11^,^12^ and capillary electrophoresis^13^,^14^ have been developed for the determination of enantiomeric citalopram. Although several chiral methods including LC^15^–^20^ and CE^21^,^22^ are available for separation of racemic citalopram, there is no report concerning enantiomeric separation of citalopram using thin layer chromatography (TLC).

In this study we develop two simple, economic and validated methods for determination of escitalopram and enantioseparation of its racemic mixture in drug substance and drug products. The fluorimetric method was based on the fluorescence spectral behavior of escitalopram in micellar systems, such as Triton^®^ X-100, Cetylpyridinium bromide; and sodium dodecyl sulfate (SDS). The fluorescence intensity of escitalopram and its racemic mixture citalopram was compared under the same experimental conditions. The method was successfully applied to the analysis of escitalopram in drug substances, drug product as well as spiked human plasma. Furthermore, the method was found to tolerate high concentrations of co-administrated and common drugs without potential interference. In addition to the fluorimetric method, TLC densitometry was proposed for stereoselective separation of (±) citalopram and determination of its enantiomer, escitalopram, using the different chiral selectors namely, brucine sulphate, chondroitin sulphate, heparin sodium and hydroxypropyl-β-cyclodextrin (HP-β-CD). The developed TLC method based on chiral mobile phase additives (CMPAs), tend to be cheap and feasible and offer a potential strategy for simultaneous separation of different chiral drugs on the same plate. The method was validated according to ICH guidelines and can become the method of choice compared to other techniques for fast routine enantiomeric analysis.

## Experimental

### Instrumentation

Waters-2525 LC system, equipped with a dual wavelength absorbance detector 2487, an auto-sampler injector and Mass Lynx v 4.1, was used. The LC column was C_18_ reverse-phase column (4.6 mm diameter × 100 mm length, 5 μm particles, phenomenex, monolithic).^1^ H-NMR spectra were recorded on Bruker Avance-400 spectrometer operating at 400 MHz. FT-IR spectrometer Avatar 360 was used. Spectrofluorimetric measurement was carried out using a Shimadzu spectrofluorimeter Model RF-1501 equipped with xenon lamp and 1-cm glass cells. Excitation and emission wavelengths were set at 242 nm and 306 nm respectively. Precoated TLC plates (10 × 10 cm, aluminum plate coated with 0.25 mm silica gel F254) were purchased from Merck Co., Egypt. Samples were applied to the TLC plates with 25 μL Hamilton microsyringe. UV short wavelength lamp (Desaga Germany) and Shimadzu dual wavelength flying spot densitometer, Model CS-9301, PC were used. The experimental conditions of the measurements were as follows: wavelength = 240 nm, photo mode = reflection, scan mode = zigzag, and swing width = 10.

### Chemicals and reagents

All chemicals used were of analytical grade if not stated otherwise. Escitalopram oxalate (Alkan Pharm Co., Egypt) certified to contain 99.60% was used as the reference standard. Cipralex containing 10 mg escitalopram oxalate per tablet (manufactured by Lund beck Co., Denmark, Batch No 2147226, Mfg. D: 2008, Exp. D: 2011) was purchased from the market. Escitalopram oxalate was extracted and purified from cipralex tablets. Citalopram was kindly supplied by Adwia Co., Egypt. Its purity was found to be 99.80% according to official HPLC method.^23^ Lecital, containing 40 mg citalopram hydrobromide per tablet (manufactured by Joswe Medical Co., Jordon) was purchased from the market. Human plasma was kindly supplied from Vacsera, Egypt. Sodium dodecyl sulfate (BHD, Egypt), brucine sulphate (BHD, Egypt), chondroitin sulfate, (Eva Co., Egypt), heparin sodium and 2-hydroxypropyl-β-cyclodextrin (Fluka, Egypt) were purchased. Trifluoroacetic acid (Aldrich, U.K), methanol and acetonitrile (Fisher Scientific, U.K.) were LC grade. Ultra pure water (ELGA, U.K.) was used.

#### Extraction of escitalopram from cipralex tablets

Ten cipralex tablets were finely powdered and transferred to a 100 mL conical flask to which 50 mL methanol was added and stirred for 20 min. The solution was filtered through whatman No. 42 filter paper. The residue was washed several times with small volume of methanol for complete recovery. The combined extract was evaporated and the pure sample was obtained by recryslallization from methanol.

#### Characterization of isolated escitalopram

The weight of escitalopram oxalate obtained by extraction and recrystalization was the same as the labeled value. Characterization of the extracted escitalopram was done using UV, TLC, LC-MS and NMR.

Absorbance spectra were recorded in methanol and TLC separation was carried out using tolueneethyl acetate-triethylmine (7:3.5:3 v/v/v) as the mobile phase.^11^ LC-MS was used for establishing the purity of escitalopram using a reverse phase C_18_ column at flow rate of 1 ml/min and acetonitrile: water:trifluoroacetic acid (60:40:0.01% v/v/v) mobile phase. Further characterization included FT-IR, 1H-NMR in deuterated methanol (CD_3_OD).

### Standard solutions

Standard stock solutions of (±)citalopram hydrobromide, and *S*-(+)escitalopram oxalate 0.05 mg mL^−1^ and 4 mg mL^−1^ were prepared by dissolving appropriate amounts of each in water and methanol for fluorimetric and TLC methods respectively. The stock solutions were subsequently used to prepare working standards in methanol. All solutions were stored in refrigerator at 4 °C.

### Synthetic mixtures

For TLC method, synthetic mixtures of escitalopram and citalopram in proportions ranging from 10%–90% were analyzed and the percentage recovery of escitalopram was calculated.

### Method development

#### Spectrofluorimetric method

1 mL of aqueous stock solution equivalent to (1.25–162.5 μg mL^−1^) of escitalopram or (1.25–125.0 μg mL^−1^) citalopram was transferred into a series of 10 mL volumetric flasks followed by 1 mL SDS (5 mmol aqueous solution). The volume was completed to the mark with methanol. The fluorescence was measured at 306 nm using 242 nm as excitation wavelength. To obtain the standard calibration graph, concentrations were plotted against fluorescence intensity and the linear regression equations were computed.

#### TLC method

Chromatograms were developed in clean, dry, paper-lined glass chambers (12 × 24 × 24 cm) pre-equilibrated with developer for 10 minutes. The TLC plates were prepared by running the mobile phase of acetonitrile-water (17:3 v/v) containing 1 mmol chiral selector to the lost front in the usual ascending way and were air-dried. For detection and quantification, 10 μL each of citalopram and escitalopram solutions within the quantification range were applied side-by-side as separate compact spots 20 mm apart and 10 mm from the bottom of the TLC plates using a 25 μL Hamilton micro syringe. The chromatograms were developed up to 8 cm in the usual ascending way using the same mobile phase omitting the chiral selectors, and were then air dried. The plates were visualized at 254 nm or by exposure to I_2_ vapor and scanned for escitalopram at wavelength 240 nm using the instrumental parameters mentioned above.

For quantitative determination of escitalopram aliquots of standard solution (4 mg mL^−1^) equivalent to 0.125–4.000 mg were transferred into 10 mL volumetric flasks and made up to volume with methanol. 10 μL of each concentration was applied on the TLC plate, air dried and scanned for escitalopram at 240 nm using the instrumental parameters mentioned above. The average peak areas were calculated and plotted against concentration. The linear relationship was obtained and the regression equation was recorded.

### Application to tablets

An accurately weighed amount of powdered tablets equivalent to 100 mg of escitalopram and citalopram were dissolved in 50 mL methanol. The solutions were stirred with magnetic stirrer for 20 min. Each solution was transferred quantitatively to a 50 mL volumetric flask, diluted to the volume with methanol, and filtered. For fluorimetric analysis, a portion equivalent to 25 mg was evaporated, transferred quantitatively to a 50 mL volumetric flask and made up to volume with water. The procedure was completed as mentioned above.

### Application to spiked human plasma

Aliquots equivalent to 0.1–0.4 mg mL^−1^ of escitalopram were sonicated with 1 mL plasma for 5 minutes. Acetonitrile (2 mL) was added and then centrifuged for 30 minutes. One milliliter of supernatant was evaporated and the procedure was completed as described above.

## Results and Discussion

In this work a simple method was used for isolation of escitalopram from its drug product rather than the published procedure.^24^ The isolated escitalopram was characterized and confirmed by different analytical techniques as mentioned above.

### Fluorimetric method

**Figure 2.**
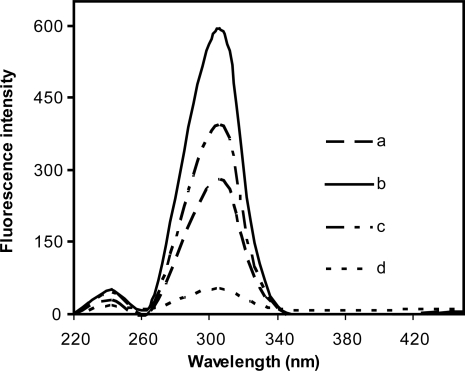
Fluorescence spectra of 10 μg mL^−1^ escitalopram oxalate **a**) in methanolic medium **b**) in 5 mM SDS micellar medium **c**) spiked plasma sample (6.6 μg mL^−1^) in 5 mM SDS micellar medium **d**) blank plasma sample.

Escitalopram solution was found to exhibit an intense fluorescence at a wavelength of 306 nm on excitation at 242 nm as shown in Figure 2. Different media such as water, methanol and ethanol were attempted. Maximum fluorescence intensity was obtained upon using methanol as diluting solvent, while water decreases the fluorescence intensity.

The effect of different surfactants on the fluorescence intensity of escitalopram was studied by adding 1 mL of each surfactant to the aqueous drug solution. CPB and Triton X-100 led to peak broadening and no effect on fluorescence intensity, while SDS caused two fold increasing in the intensity. The fluorescence intensity was stable for at least two hours.

When compared to its racemic form, escitalopram showed a lower fluorescence intensity. This is concordance with published data giving the molar absorbitivity of escitalopram as 13.630 mol^−1^cm^−1^ while that of citalopram is 15.630 mol^−1^cm^−1^.^8^ The quantum yield was found to be 0.026 for escitalopram and 0.030 for citalopram according to the following equation.^25^

Yu=Ys . Fu/Fs . As/Au

where Yu and Ys referred to fluorescence quantum yield of escitalopram and quinine sulphate, respectively; Fu and Fs represented the integral fluorescence intensity of escitalopram and quinine sulphate, respectively; Au and As referred to the absorbance of escitalopram and quinine sulphate at the excited wavelength respectively.

The method was validated by testing linearity, specificity, precision and reproducibility as presented in (Table 1).

Calibration plot was found to hold good over a concentration range of 0.125–16.25 μg mL^−1^ and 0.125–12.50 μg mL^−1^ for escitalopram and citalopram respectively. The procedure gave good reproducibility when applied to escitalopram drug substance over three concentration levels; 3.30, 6.60 and 13.30 μg mL^−1^. Whereas the specificity was proved by quantitate the studied drug in its tablet form, confirming non-interference from excipients and additives.

The results were comparable to those given by a reported method^8^ as revealed by statistical analysis adopting Student’s *t*- and *F*-tests, where no significant difference was noticed between the two methods as presented in (Table 2). The validity of the procedure was further assured by the recovery of the standard addition. The limit of detection (LOD) and the limit of quantification (LOQ) were found to be 0.017 and 0.056 μgmL^−1^ respectively.

The high sensitivity attained by the fluorimetric procedure allowed its successful application to the analysis of escitalopram in spiked human plasma. To avoid variation in background fluorescence, a simple deproteination of plasma samples with acetonitrile was performed followed by centrifugation, the clear supernatant containing escitalopram was analyzed. A calibration graph was obtained by spiking plasma samples with escitalopram in the range 3.30–16.25 μg mL^−1^. Linear regression analysis of the data gave the equation

$$\begin{array}{l} FI=37.27\;\text{C}\;+126\\ \;r=0.991\;(\text{n}=6) \end{array}$$

where *FI*is the fluorescence intensity, *C* is the concentration of escitalopram in plasma in μg mL^−1^ and *r* is correlation coefficient. The limit of detection and quantification in spiked plasma were found to be 0.17 μg mL^−1^ and 0.56 μg mL^−1^. The average recovery was 98.00% ± 2.80% RSD. The results from analysis of 5 spiked plasma samples are presented in Table 2.

The interference due to co-administrated and common drugs was investigated in mixed solutions containing 5 μg mL^−1^ escitalopram and different concentrations of an interferant. The resulting fluorescence was compared to those obtained for escitalopram only at the same concentration. Tolerance was defined as the amount of interferant that produced an error not exceeding 5% in determination of the analyte. The method was found to be selective enough to tolerate high concentration of co-administerated and common drugs. Table 3, shows the maximum tolerable weight ratio for these drugs.

The fluorimetric method offers simplicity, rapid response and the potential to be efficient for bioavailability assessments and therapeutic drug monitoring of patients treated with citalopram or escitalopram.

### TLC-densitometric method

Compared to other chromatographic techniques, TLC is a simple, economical, rapid and flexible technique allowing sensitive parallel processing of many samples on one plate. For enantiomeric separation, chiral stationary phases and mobile phase additives can be used. Brucine, chondroitin, heparin and HP-β-CD were used as chiral selectors for enantiomeric separation of different pharmaceutical compounds using TLC, LC and CE.^26^–^28^

The literature reveals that chiral recognition may occur due to formation of inclusion complexes, hydrogen-bonding, π–π interaction, hydrophobic interaction or steric repulsion.^29^ For instance, enantioselectivity using brucine arises due to the formation of two diastereomers through simple ionic interactions between racemate and chiral selector, e.g. (+)-citalopram/brucine and (−)-citalopram/brucine.^30^ The enantiomeric resolution by HP-β-CD may involve the inclusion of drug within the CD cavity relative to the comparability of sizes, shapes and hydrophibicities. Whereas steric effect derived from the anion of chondroitin sulphate contributes mainly to the interactions with drug enantiomer,^31^ the chiral discriminating capability of heparin is believed to be due to formation of a helical structure in aqueous solution.

**Figure 3.**
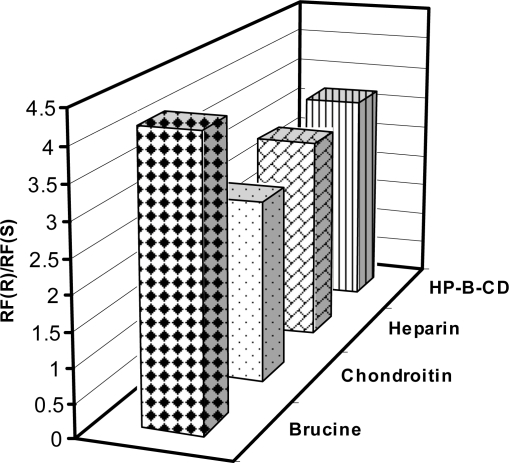
Effect of chiral selectors (1 mM) on enantiomeric resolution of racemic citalopram hydrobromide by silica gel TLC (20 μg/spot).

**Figure 4.**
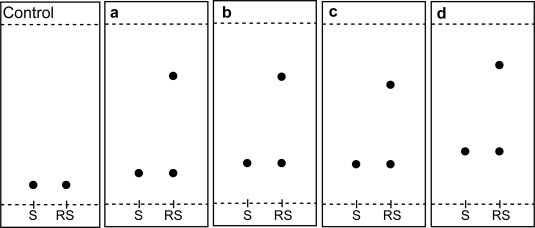
Thin layer chromatogram showing resolution of racemic citalopram hydrobromide, 20 μg/spot using different chiral selectors 1 mM, **a**) brucine sulphate, **b**) chondroitin sulfate **c**) heparin sodium, **d**) HP-β-CD; acetonitrile:water (17:3 v/v); solvent front 8 cm, 25 ± 2 ^°^C, compared with control without chiral selector.

In this work, TLC methodology was developed for separation of (±) citalopram and determination of escitalopram using different chiral selectors, the method depending on the difference in R_f_ values of (*R*)- and (*S*)- forms of (±) citalopram. The experimental conditions such as mobile phase composition, chiral selector, pH and temperature were optimized to provide accurate, precise, reproducible and robust separation. Various chiral mobile phase additives including brucine sulphate, chondroitin sulphate, heparin sodium and HP-β-CD were tested. The best resolution was achieved by using 1 mM of brucine sulphate in acetonitrile:water (17:3 v/v) as a mobile phase (Table 4). The order of enantioselectivity was found to be brucine sulphate > HP-β-CD > heparin sodium > chondroitin sulphate as shown in Figure 3. The R_f_ values were 0.17, 0.22, 0.22, 0.29 for escitalopram and 0.71, 0.70, 0.66, 0.77 for (*R*)-citalopram for the four selected chiral additives respectively as shown in Figure 4. Due to it’s lower health risks, HP-β-CD was chosen over brucine sulphate for the determination of escitalopram. We also investigated the effect of pH and temperature on resolution of racemic citalopram as they have been known to affect chiral recognition.^26^ The best conditions for discrimination of citalopram enantimers were found at pH 8.0 and 25 ± 2 °C.

### Method validation

**Figure 5.**
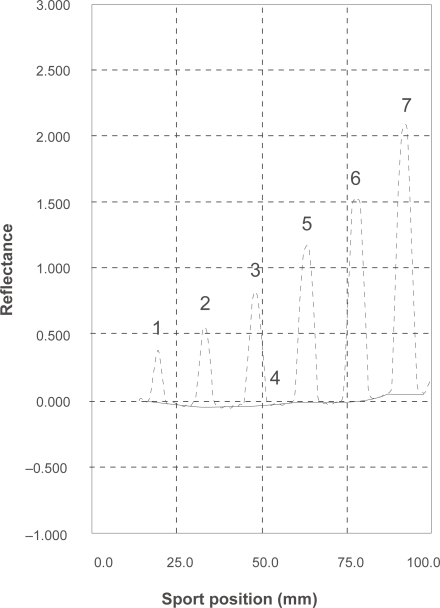
Densitometric scanning profile for TLC-chromatogram of different concentrations of escitalopram oxalate, (0.5–40 μg/spot) at 240 nm.

The method was validated according to ICH regulations by documenting its linearity, accuracy, precision, limit of detection and quantification, specificity and, robustness.^30^,^33^ The good linearity was obtained for seven concentrations in the range of 0.5– 40 μg/spot as shown in Figure 5. The accuracy based on the mean percentage of measured concentrations (n = 6) to the actual concentration is stated in (Table 1). The precision of the method was assessed by determining RSD% values of intra-and inter-day analysis (n = 9) of escitalopram over three days. Two different analysts performed intermediate precision experiments with separate mobile phase systems according to the proposed procedure. The RSD% values of the intermediate precision are less than 2% for drug substance and drug product. The LOD and LOQ were found to be 0.014 and 0.076 μg/spot respectively (Table 1). The specificity of the method was assessed by analyzing synthetic mixtures of escitalopram and citalopram in different proportions as shown in (Table 5). The conditions for this method were modified slightly with respect to mobile phase ratio, pH and temperature, the results indicating its ability to remain unaffected by small changes in the method's parameters, thus the method is considered robust.

The standard addition recoveries were carried out by adding a known amount of escitalopram to the powdered tablets at three different levels (5, 10 and 20 μg) with each level in triplicates (n = 3). The recovery percentage was evaluated by the ratio of the amount found to added. The average recovery was calculated and presented in (Table 6).

## Conclusion

The present work makes use of micelle enhanced intrinsic fluorescence of escitalopram for its determination in drug substance, commercial tablets and spiked human plasma. It was found to be selective and tolerate high concentrations of other co-administrated and common drugs. The TLC method developed was effective for enantioseparation and determination of enantiomers of citalopram. A comparative study using different chiral selectors was described with the methods being completely validated, showing satisfactory data for all validation parameters tested. Both methods offer simplicity, rapid response and economy.

## Figures and Tables

**Table 1.** Validation report on the fluorimetric and TLC-densitometric methods for the determination of escitalopram in drug substance.

**Parameters**	**Fluorimetric method**	**TLC-method**
Linearity range	0.125–16.250 μg mL^−1^	0.50–40.00 μg/spot
Regression equation		
Slope	55.556	264.240
SE of Slope	0.513	2.460
Intercept	25.466	195.730
SE of Intercept	5.377	50.470
Correlation Coefficient (r)	0.9998	0.9997
SE of estimation	8.438	102.360
Accuracy		
Mean^a^ ± RSD%	99.71 ± 1.05	100.10 ± 1.63
Precision (Mean ± RSD%)		
Intra-day^b^	99.40 ± 0.41	100.08 ± 1.38
Inter-day^b^	99.54 ± 1.08	99.70 ± 2.09
LOD	0.017	0.014
LOQ	0.056	0.046

^a^ n = 6;

^b^ n = 9.

**Table 2.** Analytical applications of fluorimetric method.

**Forms**		**Fluorimetric method**		**Reported method^a^ (8)**
Drug substance				
Mean		99.71		99.28
SD		1.05		0.81
V		1.10		0.66
SE		0.40		0.31
n		7		7
*t*-test (2.228)^b^		0.84		
*F*-test (4.53)^b^		1.67		
Cipralex tablet 10 mg/tablet, escitalopram oxalate	Recovery^c^ ± RSD%	Added μg mL^−1^	Found recovery^c^ ± RSD%	
		0.250	101.60 ± 0.85	97.30 ± 1.98
	98.90 ± 1.22	5.000	99.00 ± 1.06	
		12.500	100.9 ± 1.57	
Spiked plasma	Recovery^c^ ± RSD%			
Drug substance	98.00 ± 2.80			–
Cipralex tablet 10 mg/tablet, escitalopram oxalate	90.00 ± 3.50			

^a^ Spectrophotometric method.

^b^ Theoretical values, at P = 0.05.

^c^ Mean of four experiments.

**Table 3.** Interference study of different compounds in the determination of 5 μg mL^−1^ of escitalopram by fluorimetric method.

**Drugs**	**Tolerated interference/analyte ratio^a^ (w/w)**
1.Co-administerated	
Paroxetine HCl	100
Loratadine	100
Propranolol	20
Alprazolam	100
2.Common	
Pseudoephedrine HCl	100
Aspirin	100
Ibuprofen	50

^a^ Maximum ratio tested.

**Table 4.** Effect of mobile phase system on enantiomeric resolution of RS-citalopram and S-escitalopram (20 μg/spot), using brucine (1 mM) as chiral selector.

**Mobile phase system CH_3_CN:CH_3_OH:H_2_O**	**R_f_ values**			**R_f_(R)/R_f_(S)**
	**Pure**	**Racemic mixture**		

	***S*-(+)**	***R*-(−)**	***S*-(+)**	
16:1:3	0.22	0.80	0.22	3.64
17:0:3	0.17	0.71	0.17	4.18
20:4:0	0.26	0.75	0.26	2.88

**Table 5.** Determination of escitalopram in presence of racemic citalopram in synthetic mixtures by TLC Method.

**Sample**	**Escitalopram: citalopram (μg: μg)**	**Recovery^a^ (%)**	**RSD (%)**
1	36:4	98.00	1.48
2	16:24	99.13	0.23
3	32:8	98.33	1.66
4	4:36	98.44	1.98

^a^ All measurements were made in triplicates, 25 °C ± 2 °C.

**Table 6.** Analysis results for determination of escitalopram in cipralex tablets and application of standard addition technique by TLC method.

**Sample**	**Recovery^a^ ± RSD%**	**Standard addition**	
		**Escitalopram authentic added μg/spot**	**Found recovery^a^ ± RSD%**
1	100.52 ± 0.97	5	102.00 ± 2.09
2	101.19 ± 1.29	10	101.87 ± 1.99
3	98.45 ± 0.59	20	98.71 ± 1.07

^a^ n = 3.
